# Identification of MFGE8 and KLK5/7 as mediators of breast tumorigenesis and resistance to COX-2 inhibition

**DOI:** 10.1186/s13058-021-01401-2

**Published:** 2021-02-15

**Authors:** Jun Tian, Vivian Wang, Ni Wang, Baharak Khadang, Julien Boudreault, Khldoun Bakdounes, Suhad Ali, Jean-Jacques Lebrun

**Affiliations:** Department of Medicine, McGill University Health Center, Cancer Research Program, 1001 Decarie Blvd, Bloc E, Suite E02.6224, Montreal, QC H4A 3J1 Canada

**Keywords:** COX-2, Celecoxib, Breast cancer, TNBC, Drug resistance, MGFE8, KLK5/7

## Abstract

**Background:**

Cyclooxygenase 2 (COX-2) promotes stemness in triple negative breast cancer (TNBC), highlighting COX-2 as a promising therapeutic target in these tumors. However, to date, clinical trials using COX-2 inhibitors in breast cancer only showed variable patient responses with no clear significant clinical benefits, suggesting underlying molecular mechanisms contributing to resistance to COX-2 inhibitors.

**Methods:**

By combining in silico analysis of human breast cancer RNA-seq data with interrogation of public patient databases and their associated transcriptomic, genomic, and clinical profiles, we identified COX-2 associated genes whose expression correlate with aggressive TNBC features and resistance to COX-2 inhibitors. We then assessed their individual contributions to TNBC metastasis and resistance to COX-2 inhibitors, using CRISPR gene knockout approaches in both in vitro and in vivo preclinical models of TNBC.

**Results:**

We identified multiple COX-2 associated genes (TPM4, RGS2, LAMC2, SERPINB5, KLK7, MFGE8, KLK5, ID4, RBP1, SLC2A1) that regulate tumor lung colonization in TNBC. Furthermore, we found that silencing MFGE8 and KLK5/7 gene expression in TNBC cells markedly restored sensitivity to COX-2 selective inhibitor both in vitro and in vivo.

**Conclusions:**

Together, our study supports the establishment and use of novel COX-2 inhibitor-based combination therapies as future strategies for TNBC treatment.

**Supplementary Information:**

The online version contains supplementary material available at 10.1186/s13058-021-01401-2.

## Background

Triple negative breast cancers (TNBCs) account for around 15% of all breast cancers and are pathologically characterized by the negative expression of estrogen receptor (ER) and progesterone receptor (PR) as well as the aberrant expression of human epidermal growth factor receptor 2 (HER2) [1]. TNBCs exhibit poor prognosis compared with other breast cancer subtypes due to their aggressive clinical features and lack of specific molecular targets [2]. TNBC treatments using chemotherapy and radiotherapy show limited therapeutic benefits, with the majority of patients still at high risk of relapse and development of distant metastasis [3]. Therefore, efforts are needed to develop new therapeutic options with long-lasting clinical responses for these deadly tumors.

Cyclooxygenase 2 (COX-2) is a key enzyme that catalyzes prostaglandins formation from arachidonic acid and is aberrantly expressed in various types of cancers, including those of the breast [4–6]. COX-2 over-expression is found in 40% of invasive breast carcinoma cases and is associated with poor prognosis and tumor progression [7, 8]. Several studies using pre-clinical models have demonstrated the involvement of COX-2/Prostaglandin E2 (PGE2) pathway in various steps during breast cancer progression, including primary tumor growth [9], metastasis [10, 11], angiogenesis [12], and immune evasion [13]. Moreover, we recently found COX-2 to be highly expressed in TNBC tumors and its expression to correlate with poor overall survival (OS) and distant metastasis-free survival (DMFS) rates [14]. We further showed that COX-2 regulates the self-renewal capacity and expansion of breast cancer stem cells, highlighting COX-2 as a very promising therapeutic target for TNBCs [14].

Selective COX-2 inhibitors (i.e., celecoxib; brand name Celebrex) are being used to treat patients with osteoarthritis and adult rheumatoid arthritis. Their safe use has been further illustrated by low associated risk factors for gastrointestinal and cardiovascular adverse events, compared with nonsteroidal anti-inflammatory drugs (NSAIDs) [15]. Interestingly, several preclinical studies in breast cancer showed the use of selective COX-2 inhibitors (celecoxib, rofecoxib, etodolac) could efficiently block breast tumor growth and metastasis [16–22]. Early phase I/II neoadjuvant trials using combined celecoxib and aromatase inhibitors in the treatment of locally advanced and metastatic breast cancers demonstrated reductions in breast tumor size and area [23, 24]. However, a recent phase III, multicenter, double-blind, randomized trial of celecoxib vs placebo in primary breast cancer patients (REACT trial) showed no benefit in delaying time to progression or overall survival [25]. These findings highlight the importance of addressing the underlying mechanisms that likely contributed to resistance to COX-2 inhibition in breast cancer. Thus, to address this unmet clinical need, a better understanding of the molecular mechanisms and genes contributing to celecoxib sensitivity/resistance in breast cancer is clearly warranted. Identification of such genes will allow for refined, more specific choice of biomarkers and patient stratification, ultimately leading to better therapeutic outcomes for TNBC patients.

With the ever-increasing release of large breast cancer patient datasets, a wealth of patient data, including tumor genomic and transcriptomic profiles as well as patient clinical information have become available for data mining. In this study, we employed a systematic in silico approach to identify COX-2-associated genes and their clinical value in TNBC patients. This led us to define 10 candidate genes with high potential to (1) promote TNBC tumorigenesis and (2) resistance to COX-2 inhibitors. Further functional in vitro and in vivo validation of these candidate genes using CRISPR/Cas9-mediated genomic deletion approaches, highlighted several of these genes (*TPM4*, *RGS2*, *SERPINB5*, *MFGE8*, *KLK5*, *ID4*) as potent regulators of lung colonization in preclinical TNBC model. Furthermore, we found *MFGE8*, *KLK5*, and *KLK7* knockout (KO) to restore celecoxib sensitivity in TNBC cells leading to marked reductions in primary tumor growth. Taken together, these results provide important rationale for developing COX-2 inhibitor-based combination therapies for breast cancer patients, aiming at increasing/restoring the effectiveness of COX-2 inhibitors in breast cancer patients.

## Methods

### Selection of patient data

The TCGA data used in this study are derived from Breast Invasive Carcinoma (TCGA, provisional) dataset comprising 1108 samples [26, 27]. TNBC samples were manually selected from the dataset based on the IHC status of ER, PR, and HER2. Patients with a COX-2 mRNA *z*-score greater than + 1 (COX-2 high) or less than − 0.25 (COX-2 low) compared with the overall distribution were selected for comparison using cBioPortal for Cancer Genomics online application (https://www.cbioportal.org/). Genome-wide RNA-sequencing data of these patients were then downloaded from the NIH GDC data portal (https://portal.gdc.cancer.gov/). In all cases, the normalized Fragments Per Kilobase of transcript per Million mapped reads (FPKM) values of more than 67,000 genes were downloaded and used for further data analysis.

### Differential expression gene analysis

Human mRNA expression data were loaded for each patient into a single spreadsheet and uploaded to the GenePattern web software suite run by the Broad Institute. Patient data were analyzed for differential gene expression using the ComparativeMarkerSelection tool comparing COX-2-high and COX-2-low patients. This tool uses a moderated *t* test to make pairwise comparisons within the dataset and select for differentially enriched genes. The genes were ranked based on their *t*-test value and several filters (fold change >1.5, *p* value <0.05, *t*-test >2 or <−2, FDR <0.35) were applied to further select the significant differentially expressed genes. The resulted top 103 differentially expressed genes were then hierarchically clustered using the HierarchicalClustering tool on the GenePattern web application. Pairwise average linkage clustering with Pearson’s correlation coefficient was used to measure the similarity.

### Selection of candidate genes

The 43 DEGs enriched in COX-2-high TNBC patients were analyzed for their genetic alteration rate (gene copy number amplification, mRNA upregulation) in TCGA-BRCA TNBC dataset (*n* = 116) and Metastatic Breast Cancer dataset (*n* = 180) using the cBioPortal for Cancer Genomics online application. These 43 DEGs were also analyzed for their gene expression levels across various breast cancer molecular subtypes (Luminal A, Luminal B, HER2-enriched, Basal, Normal-like) as well as various sample types (primary tumor, metastatic tumor, solid normal tissue) in TCGA-BRCA dataset (*n* = 1247) using the UCSC Cancer Genomics Browser. Breast Cancer Gene-Expression Miner v4.0 (bc-GenExMiner v4.0) online platform comprising 5696 breast cancer samples was used to assessed for the gene expression levels of the 43 DEGs in TNBC versus non-TNBC samples. Kaplan-Meier plotter database comprising 241 basal-like breast cancer samples was used to evaluate the association between the DEGs mRNA levels and patient outcomes including overall survival (OS) and distant metastasis-free survival (DMFS).

### Identification of genes associated with COX-2 inhibitor resistance

The information of 38 breast cancer cell lines' sensitivity to COX-2 selective inhibitor, valdecoxib, was obtained from the Cancer Therapeutics Response Portal v2 (CTRP v2). The cell lines were ranked based on their valdecoxib EC50 value and categorized into more -sensitive cell lines (*n* = 18) and less-sensitive cell lines (*n* = 19). Then the DEG expression data in breast cancer cell lines were downloaded from the Cell Line Gene Expression (CCLE) dataset and each of the DEGs was analyzed for mRNA expression in valdecoxib-sensitive cell lines and valdecoxib-resistant cell lines.

### Cell culture and generation of celecoxib-resistant cells

Human breast cancer cell lines MDA-MB-231 and SUM159 were cultured as previously described [14]. We generated two MDA-MB-231 variant cell lines enriched of celecoxib-resistant cells, using increasing concentrations (40 μM and 80 μM) of celecoxib (pZ0008-5MG, Sigma). Selection pressure was maintained for 3 weeks under these cell culture conditions. At end point, and as a proof-of-principle, COX-2 expression was assessed at both mRNA and protein levels to verify the proper COX-2 increase normally observed in Cox-2-resistant cells [28].

### CRISPR-Cas9 sgRNA cloning

Different scrambled sgRNAs and sgRNAs that target COX-2 and 10 candidate genes were cloned into the lentiCRISPR v2 backbone (Addgene plasmid # 52961) individually. All steps were performed according to the protocol provided by Feng Zhang’s lab. Briefly, lentiCRISPR v2 plasmid was first digested and phosphorylated with BsmBI and then gel purified. Two oligos of each sgRNA were phosphorylated and annealed to each other using T4 Ligation Buffer. Next, diluted oligos and BsmBI digested lentiCRISPR v2 plasmid were ligated and transformed into Stbl3 bacteria. PCR was performed to confirm the insertion of oligos in the backbone plasmid. Sequences of sgRNAs are shown in Supplementary Table S1.

### Lentivirus production and infection

LentiCRISPR v2 plasmids containing scrambled and different sgRNAs sequences were co-transfected into HEK293 cells with the packaging plasmids (psPAX2 and Pmd2.g). Transfection was performed using Opti-MEM (Invitrogen) and bPEI (Sigma). 48 h following transfection, cell culture medium containing lentiviruses were collected. To generate stable gene knockout cell line, MDA-MB-231 cells and SUM159 cells were cultured to 50% confluence and then infected with lentiviruses using 8 μg/ml polybrene. Then, 24 h later, 2 μg/ml of puromycin was added to the medium to select stable cells for a minimum of 1 week.

### Surveyor assay

In total, 200,000 MDA-MB-231 and SUM159 stable KO cells were used for Surveyor assay. All experiments were performed using GeneArt® Genomic Cleavage Detection Kit (life technologies) according to the manufacturer’s protocol. Briefly, DNA loci where the gene-specific double-strand breaks occur were PCR amplified. Then these PCR products were denatured and re-annealed so that the mismatches were generated. Next the mismatches were cleaved by Detection Enzyme and detected by gel electrophoresis. Cleavage efficiency was calculated using the following equation: Cleavage efficiency = 1 − [(1 − fraction cleaved) ½]; fraction cleaved = sum of cleaved band intensities/ (sum of the cleaved and parental band intensities). Sequences of PCR primers are shown in Supplementary Table S2.

### Distant metastasis mouse model

All animal experiments were conducted in accordance with protocols approved by the McGill University Health Center. Scrambled and candidate gene sgRNA transfected MDA-MB-231 cells (and SUM159 cells) (1 × 10^6^ cells/mouse) were injected into the tail vein of 6-week-old female NOD SCID IL2gammaR knockout (NSG) mice (4 mice per group). Three weeks post injection, mice were sacrificed, and the lungs were collected and fixed in 10% formalin. The lung tissues were then embedded in paraffin, sectioned, and stained with hematoxylin and eosin (H&E).

### Quantification of H&E staining

The mean percentage of total lung involvement based on a visual scoring was performed by two pathologists. A variety of patterns were observed in tumor cells including a small nodular, infiltrative, and solid pattern. Where applicable, the largest size of nodule in lung parenchyma was measured microscopically.

### Orthotopic xenograft mouse model

The scrambled and candidate gene KO cells generated from MDA-MB-231 cell line were resuspended in serum-free medium and Corning™ Matrigel™ in 1:1 ratio and then transplanted in the mammary gland of 6-week-old female NSG mice (1 × 10^6^ cells per mouse, 8 mice per group). When the tumor size reached 150–200 mm^2^, each mice group was randomly subdivided into two groups and treated with either vehicle or celecoxib (7.5 mg/kg/day) through intraperitoneal (IP) injection for up to 3 weeks. Primary mammary tumor size was measured using a caliper (number) times every week and determined according to the formula: (4/3) × π × (Length/2) × (width/2)^2^. The mice were sacrificed when control tumors reach max authorized volume (2.5 cm^3^).

### Sulforhodamine B (SRB) assay

SRB assay was used to measure growth inhibition in cells. MDA-MB-231 cells (and SUM159 cells) (CRISPR scrambled and stable KO cells) were grown in DMEM complete medium (and F12 HAM’s complete medium, respectively) (2500 cells/well) in a 96-well plate and allowed to attach for 24 h. The cells were then treated with a dose range of celecoxib for 96 h. After treatment, the cells were fixed with 50% trichloroacetic acid (TCA) for 2 h at 4 °C, rinsed with water 4 times, stained with 0.4% SRB for 1 h and rinsed with 1% acetic acid. After air dry overnight, the SRB dye was solubilized with 10 mM Tris base and the plates were read at 490 nm using a microplate reader. The results were analyzed and graphed using GraphPad Prism 6.0 (GraphPadSoftware, Inc., San Diego, CA).

### Prestoblue assay

MDA-MB-231 (and SUM159 cells) were seeded into 96-well plates with black bottom (2500/well). Then, 24 h later, cells were treated with a dose range of celecoxib for 96 h and then incubated with PrestoBlue™ Cell Viability Reagent (Thermo Fisher Scientific) for 40 min at 37 °C/5% CO_2_. Fluorescence measurements (excitation 535 nm, emission 615 nm) were then taken on the 96-well plates and the fluorescence values were recorded and analyzed. Since the fluorescence values have a linear correlation with the cell numbers, the data were used to calculate the percentage of cell viability inhibition following celecoxib treatment.

### Western blot

Western blot analysis was performed as previously described [14]. Briefly, human breast cancer cells were lysed in Tris lysis buffer. Lysates containing total protein were separated by SDS-PAGE and transferred to nitrocellulose membrane. COX-2 protein levels were detected using rabbit monoclonal COX-2 antibody (Cell Signalling). Mouse monoclonal β-tubulin antibody (Santa Cruz Biotechnology) was used as loading controls. Each protein was detected using Clarity™ ECL western blotting substrate from Bio-Rad.

### Statistics

Student’s *t* test or one-way ANOVA was used to evaluate significance between groups. At least three independent experiments were performed and *P* < 0.05 was considered significant.

### Study approval

All experimental protocols and procedures were performed in accordance to McGill University regulations. All experimental protocols and procedures were approved by McGill University.

## Results

### Identification of COX-2 associated genes in TNBC tumors

**Fig. 1:**
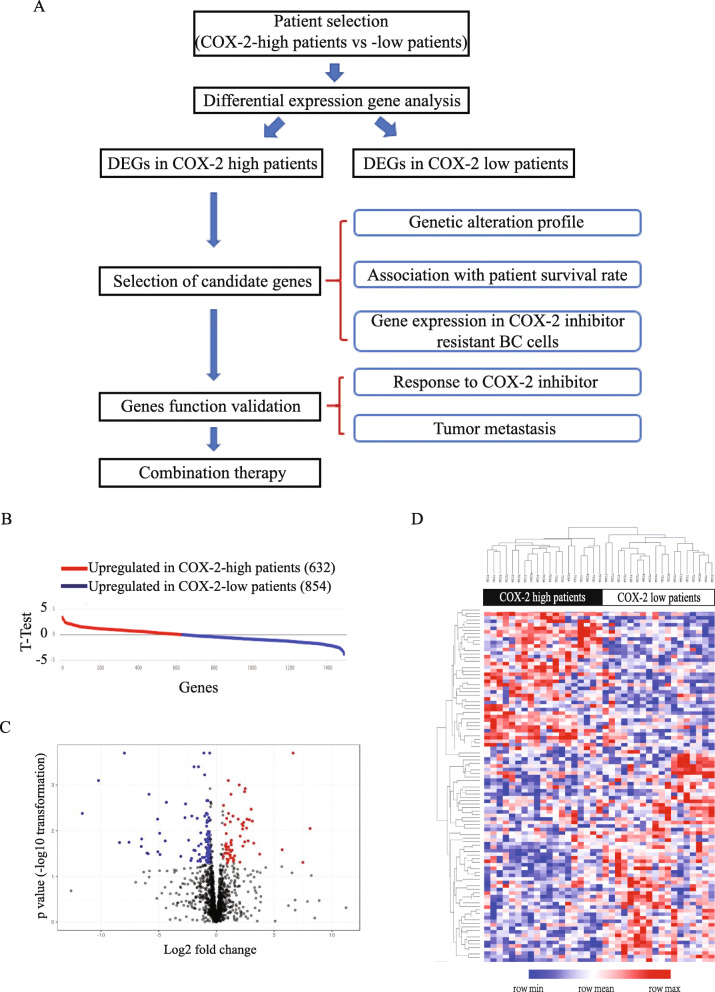
Identification of COX-2 associated genes in TNBC tumors. **a** Illustration of our data mining strategy. **b**, **c** *T*-test value and volcano plot of differential expressed genes in COX-2-high patients (*n* = 18) versus COX-2-low patients (*n* = 19). **d** Heatmap clustering analysis on the selected COX-2-high and COX-2-low patients using the 103 differentially expressed gene signature. Red and blue colors indicate high and low gene expression, respectively

A systematic data mining approach, using publicly available patient databases containing transcriptomic and genomic profiles as well as clinical data was developed to identify COX-2 associated genes with a potential to promote tumorigenesis and to regulate COX-2 inhibitor responses in TNBC. As illustrated in Fig. 1a, we initially used 116 TNBC patient samples (Supplementary Table S1) from TCGA-BRCA dataset and subdivided them based on their COX-2 mRNA expression levels (COX-2-high vs COX-2-low patients) using cBioPortal for Cancer Genomics online application (https://www.cbioportal.org/) [29, 30]. The top 15% COX-2-high patients samples (*n* = 18, *z*-score greater than 1) and the bottom 15% of COX-2-low patients samples (*n* = 19, *z*-score less than − 0.25) were then selected and assessed for differential expression gene (DEG) analysis using GenePattern web software [31]. DEG analysis was carried out via moderated *t* test on patient RNA-sequencing data to determine which genes were most differentially expressed in each patient group. Following analysis, genes were ranked by *t*-test value—the standardized mean difference in gene expression between each patient group. As shown in Fig. 1b, we found genes with a positive *t*-test value to be more highly expressed in the COX-2-high patient group, while genes with a negative *t*-test value were more highly expressed in the COX-2-low patient group. We also applied several filters to further select the significant DEGs. At a specified significance level (fold change >1.5, *p* value <0.05, *t*-test >2 or <−2, FDR <0.35), 43 and 60 genes were differentially enriched in COX-2-high patient and COX-2-low patient groups, respectively (Fig. 1c and Fig.S1). We next performed unsupervised hierarchical clustering analysis on the selected COX-2-high and COX-2-low patients using the 103 differentially expressed gene signature. As expected, the patient samples were categorized into 2 main clusters, which perfectly matched the pre-selected 18 COX-2-high patients and 19 COX-2-low patients (Fig. 1d), indicating that the 103 DEGs can clearly distinguish the COX-2-high and COX-2-low patient groups, thus validating our DEG selection method.

### Identification of COX-2-associated genes that show genomic alterations and poor prognostic value in TNBC

**Fig. 2:**
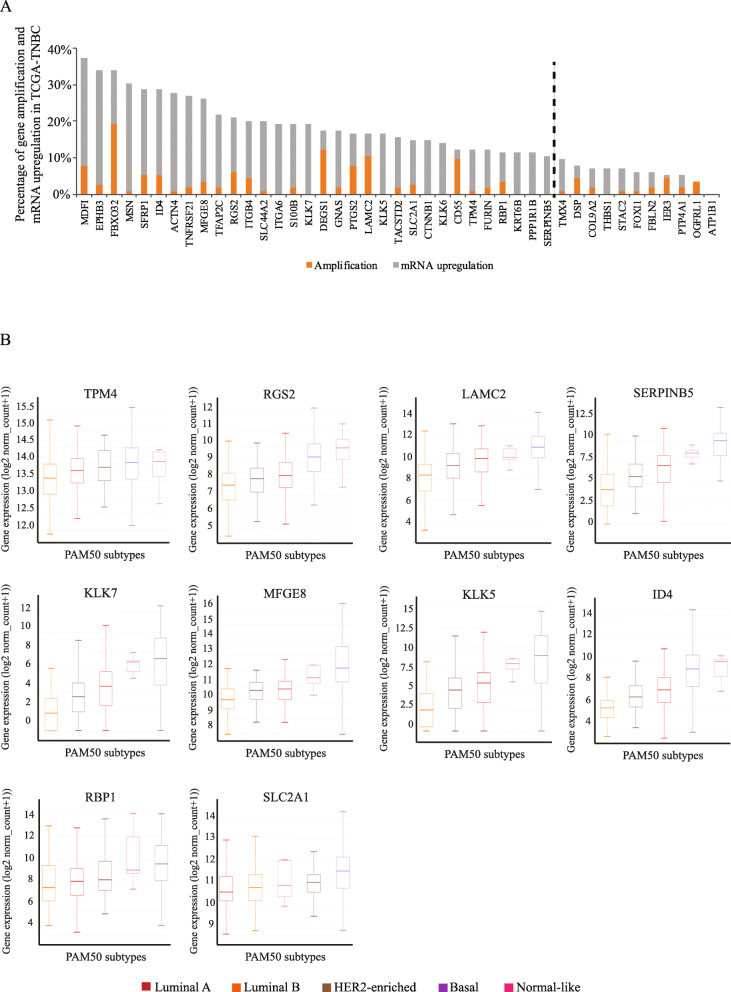
COX-2-associated genes with high genetic alteration rate and expression in aggressive breast cancer. **a** Percentage of gene copy number amplification (orange) and mRNA upregulation (gray) of each of the 43 DEGs in TCGA-BRCA TNBC patient cohorts (*n* = 116). Genes to the left of the dashed lines were considered as highly amplified and expressed. **b** mRNA expression levels of final selected 10 candidate genes in various PAM50 breast cancer subtypes from the TCGA-BRCA dataset (*n* = 1247)

To then identify COX-2-associated genes that may have similar or parallel function to COX-2 in promoting tumorigenesis, we first focused on the 43 DEGs enriched in COX-2-high patient group. We performed an unbiased and comprehensive in silico analysis of these DEGs in multiple large breast cancer patient cohorts. We examined the genetic alteration rate (gene copy number amplification, mRNA upregulation) of each of the 43 DEGs in TCGA-BRCA TNBC patient cohorts (*n* = 116) and Metastatic Breast Cancer patient cohorts (*n* = 180) using the cBioPortal for Cancer Genomics online application. As shown in Fig. 2a, the genetic alteration rate of each gene was calculated as the sum of the percentage of patients with each gene copy number amplification (copy number status: + 2) and mRNA upregulation (*z*-score greater than + 1) and was used to rank the 43 DEGs in TCGA-BRCA TNBC patient cohorts. Genes that are altered in more than 10% of the patients were considered as gene list 1 (GL1, 32 genes) (Table 1). Similarly, the 43 DEGs were also ranked based on their gene copy number amplification rate in the Metastatic Breast Cancer patient cohorts (Fig.S2A), and genes that were found amplified in more than 1% of the patients were included in gene list 2 (GL2; 36 genes) (Table 1). Thus, genes identified in GL1 and GL2 lists have the highest potential to be involved in tumorigenesis and metastasis compared to the rest of the 43 DEGs.

**Table 1:** Gene lists based on various selection criteria from the 43 DEGs in COX-2 high patients

Selection criteria	High genetic alteration in TCGA-TNBC patients (GL1)	High genetic alteration in MBC patients (GL2)	High mRNA expression in PAM50 basal BC patients (GL3)	High Mrna expression in TNBC patients (GL4)	High expression correlates with poor survival outcomes (GL5)	High expression in COX-2 inhibitor-resistant BC cell lines (GL6)
**Gene symbol**	MDFI EPHB3 FBXO32 MSN SFRP1 ID4 ACTN4 TNFRSF21 MFGE8 TFAP2C RGS2 ITGB4 SLC44A2 ITGA6 S100B KLK7 DEGS1 GNAS PTGS2 LAMC2 KLK5 TACSTD2 SLC2A1 CTNNB1 KLK6 CD55 TPM4 FURIN RBP1 KRT6B PPP1R1B SERPINB5	FBXO32 PPP1R1B THBS1 LAMC2 STAC2 ATP1B1 DEGS1 IER3 OGFRL1 GNAS TFAP2C CD55 SFRP1 ITGB4 MFGE8 EPHB3 ACTN4 RGS2 PTGS2 SLC2A1 COL9A2 DSP MDFI PTP4A1 TNFRSF21 ID4 FURIN CTNNB1 KLK7 TPM4 TACSTD2 FBLN2 SERPINB5 KLK5 KLK6 RBP1	TPM4 TNFRSF21 RGS2 TFAP2C ITGA6 COL9A1 SFRP1 FURIN LAMC2 TACSTD2 MSN S100B FBXO32 STAC2 DSP KRT6B SERPINB5 ITGB4 FOXI1 EPHB3 MDFI KLK7 OGFRL1 MFGE8 ACTN4 KLK5 SLC44A2 KLK6 ID4 RBP1 PTP4A1 TMX4 SLC2A1 GNAS	TPM4 TNFRSF21 RGS2 TFAP2C ITGA6 SFRP1 FURIN LAMC2 CD55 MSN S100B FBXO32 STAC2 KRT6B FBLN2 SERPINB5 ITGB4 EPHB3 MDFI KLK7 OGFRL1 MFGE8 ACTN4 KLK5 SLC44A2 KLK6 ID4 RBP1 TMX4 SLC2A1 GNAS	TPM4 RGS2 TFAP2C ITGA6 ATP1B1 LAMC2 CD55 TACSTD2 CTNNB1 KRT6B SERPINB5 IER3 ITGB4 KLK7 MFGE8 KLK5 ID4 RBP1 PTP4A1 SLC2A1	TPM4 RGS2 COL9A2 SFRP1 LAMC2 FBLN2 SERPINB5 KLK7 MFGE8 KLK5 ID4 RBP1 SLC2A1

Next, we analyzed the mRNA expression levels of the 43 DEGs in various PAM50 breast cancer subtypes from the TCGA-BRCA dataset using the UCSC Cancer Genomics Browser (https://genome-cancer.ucsc.edu). Genes that displayed the highest expression level in basal BC compared with the other subtypes (Luminal A, Luminal B, HER2-enriched, Normal-like) were selected and included in genes list 3 (GL3; 34 genes) (Table 1 and Fig. 2b). In parallel, we also compared the candidate genes expression in TNBC patients versus non-TNBC patients from a large breast cancer patient cohort (*n* = 5696) using the Breast Cancer Gene-Expression Miner v4.0 (bc-GenExMiner v4.0) online platform. Genes that are significantly highly expressed in TNBCs in comparison with non-TNBCs were identified as gene list 4 (GL4; 30 genes) (Table 1 and Fig.S2B). These COX-2-associated genes in GL3 and GL4 have similar expression patterns as COX-2 across different BC subtypes and therefore are likely to exhibit parallel and/or to play a role in COX-2-mediated tumorigenesis.

We also examined the association between the expression level of each DEG and survival outcomes of basal subtype of breast cancer patients, using publicly available Kaplan-Meier plotter online application (http://kmplot.com/analysis/) [32]. We selected genes whose high expression significantly associated with poor overall survival (OS) and distant metastasis-free survival (DMFS) rates and included these genes in gene list 5 (GL5; 20 genes) (Table 1 and Fig.S3). Genes that correlate with poor prognosis in breast cancer patients are more likely to play a role in tumorigenesis and as such can be used as prognostic markers and survival outcome indicators.

### Identification of COX-2-associated gene signature that correlate with resistance to COX-2 inhibitors

**Fig. 3:**
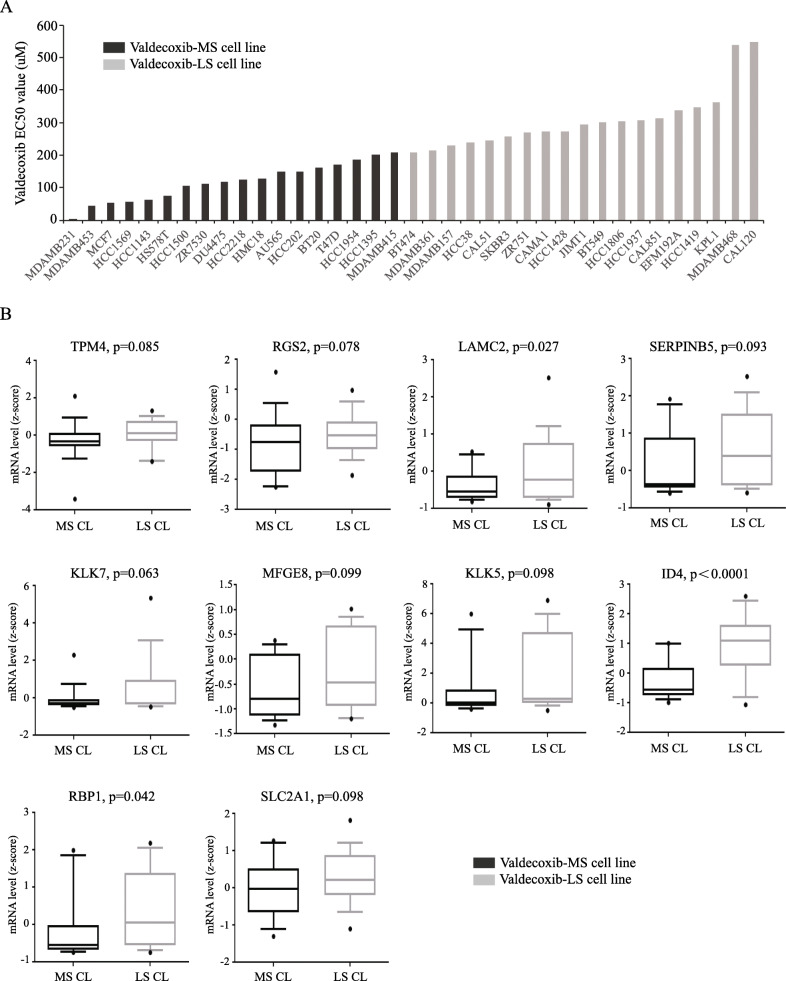
Expression of 10 candidate genes in BC cell lines with various sensitivities to COX-2 inhibitor. **a** Thirty-seven breast cancer cell lines with various responses to valdecoxib based on their EC50 values. **b** mRNA expression levels of 10 candidate genes in valdecoxib-MS BC cell lines versus valdecoxib-LS BC cell lines

Although COX-2 inhibitors may attenuate breast tumor growth in preclinical models [16–22] and early phase I/II neoadjuvant trials, when used in combination with aromatase inhibitors [23, 24], the recent phase III, multicenter, double-blind, randomized REACT trial of celecoxib vs placebo showed no benefit in delaying time to progression or overall survival in primary breast cancer patients [25]. These results underscore the existence of genes/mechanisms that likely contribute to COX-2 inhibitor resistance in breast cancer. Because expression of the 43 DEGs is the highest in the COX-2-high patient group, it is plausible that they may also modulate or affect TNBC response to COX-2 inhibitors. We thus, assessed their expression levels in cell lines with various sensitivities to COX-2 inhibitor. For this, we accessed the cancer therapeutics response portal v2 (CTRP v2) resulting from 37 breast cancer cell lines treated with the COX-2 selective inhibitor, valdecoxib (http://portals.broadinstitute.org/ctrp/). Cell lines were ranked based on their EC50 value for valdecoxib: the 18 cell lines with the lowest EC50 value were classified as more sensitive (MS) cell lines while the other 19 cell lines with the highest EC50 values were labeled as less-sensitive (LS) cell lines (Fig. 3a). Next, we analyzed the mRNA expression levels of the 43 DEGs in the valdecoxib-MS and valdecoxib-LS cell lines using the Cell Line Gene Expression (CCLE) dataset in the cBioPortal online application. Those genes that are highly expressed in the valdecoxib-LS cells were selected as gene list 6 (GL6; 13 genes) (Table 1 and Fig. 3b) and identified as potential causal factors or contributors to COX-2 inhibitor resistance.

Based on the above gene selection criteria, we next cross-referenced all gene lists (GL1-GL6) and found 10 overlapping genes (*TPM4*, *RGS2*, *LAMC2*, *SERPINB5*, *KLK7*, *MFGE8*, *KLK5*, *ID4*, *RBP1*, *SLC2A1*) that show gene alteration/amplification in TNBCs, are highly expressed in aggressive BCs and COX-2 inhibitor-LS BC cell lines, and are associated with poor BC patient outcomes. We also assessed the mRNA expression levels of these 10 genes across various sample types (primary tumor, metastatic tumor, solid normal tissue) in TCGA dataset. The analysis showed that 7 out of 10 genes displayed highest expression in solid normal tissue (Fig. S4). However, some of these genes were not detectable in most of the normal samples; thus, the comparison remains inconclusive. Furthermore, we also found expression of 9 out of these 10 genes to strongly correlate with COX-2 expression (Pearson’s correlation coefficient >0.2, *p* < 0.05) in TCGA TNBC patients even though they are located at different chromosome regions (Fig.S5). We thus reasoned that these genes have a high probability to function with or in parallel to COX-2 in promoting primary tumor formation and metastasis in TNBCs as well as in contributing to BC resistance to COX-2 inhibitors.

### Identification of COX-2-associated candidate genes in regulating TNBC metastasis

**Fig. 4:**
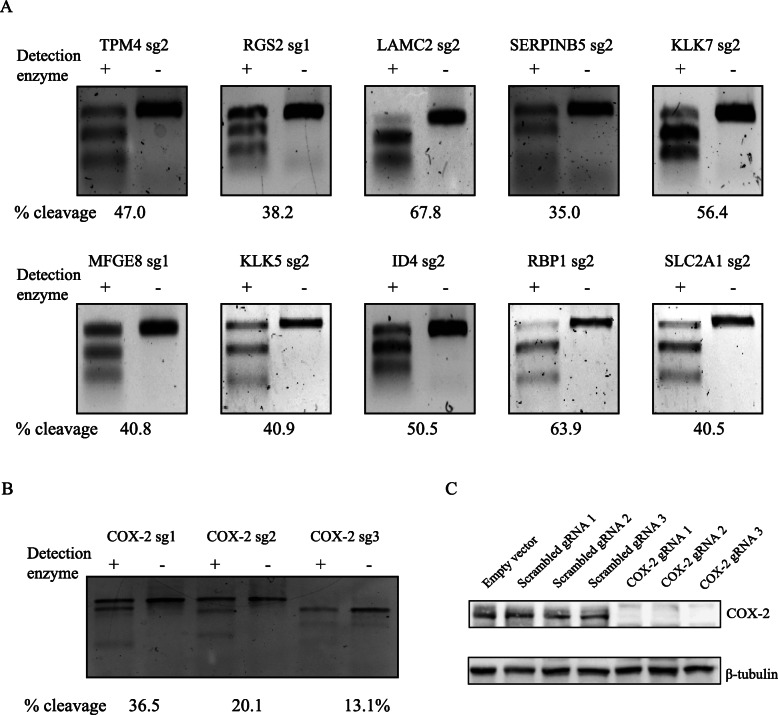
CRISPR knock out of COX-2 and 10 candidate genes in TNBC cells. **a**, **b** MDA-MB-231 cells were infected by lentivirus containing various gRNA sequences targeting 10 candidate genes (**a**) and COX-2 (**b**), and then subjected to Surveyor nuclease assay. Cleavage efficiency for each KO was calculated. **c** COX-2 KO in MDA-MB-231 cells was validated by western blot

To investigate the potential contributions of the 10 shortlisted candidate genes toward breast cancer lung metastasis, we first used CRISPR-Cas9 technology to individually knock out these 10 genes in a TNBC cell line (MDA-MB-231, derived from the pleural effusion of a metastatic breast cancer patient [33]). To ensure optimal gene disruption, we used two specific guide RNAs (gRNAs) targeting distinct genomic sites for each candidate gene as well as scrambled gRNAs as negative controls and sgRNAs targeting COX-2 as positive controls. Specific gRNAs were sub-cloned into the lentiCRISPR v2 vector and delivered into the MDA-MB-231 cell line through lentiviral infection. The presence of proper indel mutations was assessed using Surveyor nuclease assay and the cleavage efficiency was calculated to select the most efficient sgRNAs (Fig. 4a, b). Western blot analysis confirmed the efficacy of the KOs, showing complete loss of COX-2 protein expression in all 3 specific COX-2 KO stable cell lines, compared to scrambled gRNA-transfected cells (Fig. 4c).

**Fig. 5:**
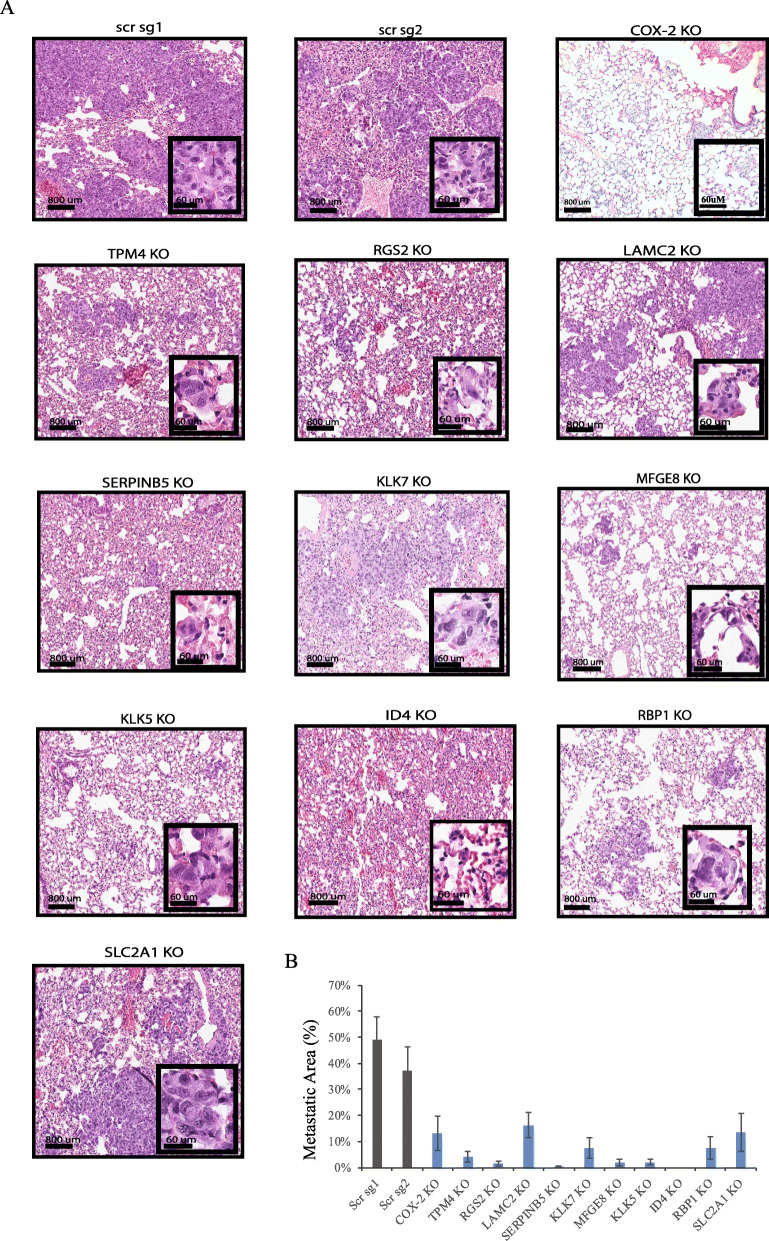
Identification of COX-2 associated genes regulating breast tumor lung metastasis. **a** H&E staining of the lung tissues from NSG mice injected with MDA-MB-231 control (scrambled) cells, COX-2 KO cells, and each of the 10 candidate genes KO cells. **b** Percentage of metastatic area of the control and KO tumors was calculated by two pathologists

As COX-2 is known to contribute to tumor progression and high COX-2 expression is found in invasive breast cancer, we next sought to assess the role and implication of the 10 COX-2-associated genes in TNBC metastasis, using a preclinical model of breast cancer lung colonization (tail vein injection). CRISPR KO MDA-MB-231 cell lines were inoculated into NOD SCID IL2gammaR (NSG) mice (1 × 10^6^ cells per mouse) through tail vein injection to allow for the seeding of the cancer cells to the lung. Three weeks following the cancer cell inoculation, lung tissues of the mice were collected and assessed for the presence of metastatic loci through hematoxylin and eosin (H&E) staining. As expected, the mice injected with scrambled MDA-MB-231 cells developed large areas of lung metastases, while the mice injected with COX-2 KO cells formed only few micrometastases (Fig. 5a, b). Consistent with the previous reports [11, 22], these data demonstrate the role of COX-2 in promoting BC metastasis in vivo. Interestingly, when assessing the metastasis-promoting effects of the 10 candidate genes, we found sgRNAs targeting all ten genes to reduce the lung metastatic area by variable extent as compared to controls (scrambled sgRNAs), with the most significant effects mediated by the deletion of *TPM4*, *RGS2*, *SERPINB5*, *MFGE8*, *KLK5*, and *ID4* (~ 90% reduction). Similarly, individual knockout of *LAMC2*, *KLK7*, *RBP1*, and *SLC2A1* also reduced metastatic colonization of the lungs (60–80% reduction) (Fig. 5a, b). These results indicate that these genes have the capacity to promote breast cancer cell colonization to the lung at a single-gene level and, thus, could serve as biomarkers and therapeutic targets for metastasis in TNBC. These results also validated our in silico analysis strategy in identifying COX-2-associated genes that promote metastasis.

### Knock-out of COX-2-associated genes LAMC2, MFGE8, KLK5, KLK7, and SLC2A1 restore sensitivity to celecoxib in TNBC

**Fig. 6:**
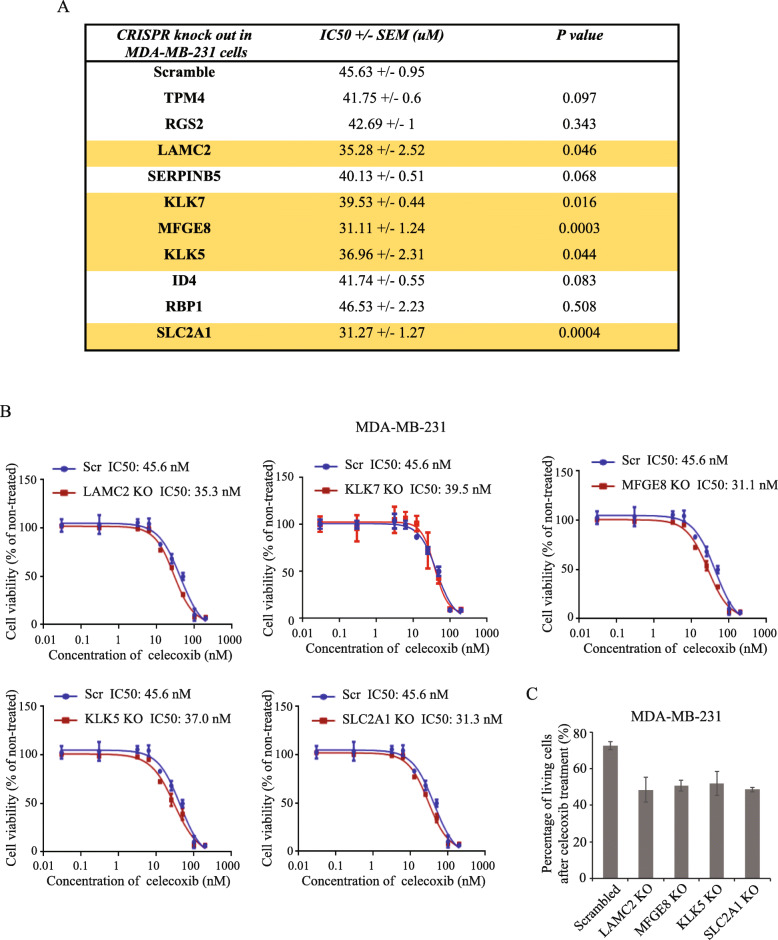
Identification of COX-2 associated genes contributing to COX-2 inhibitor resistance in TNBC cells. **a** MDA-MB-231 control and KO cell lines were treated with a dose rage of celecoxib for 4 days and subjected to cell viability test using SRB assay. IC50 values of control and each KO cells were indicated. **b** Celecoxib dose response curve in MDA-MB-231 control, LAMC2, KLK7, MFGE8, KLK5, and SLC2A1 KO cells. **c** MDA-MB-231 control, LAMC2, MFGE8, KLK5, and SLC2A1 KO cells were treated with 50 μM celecoxib for 4 days and then subjected to Prestoblue cell viability assay. Percentage of living cells after celecoxib treatment was calculated

Given that our top 10 selected genes are highly expressed in COX-2 inhibitor-resistant breast cancer cells from the CTRP dataset, this prompted us to investigate their role in mediating drug resistance in TNBC cells. The CRISPR KO cell lines generated above were treated or not with a dose range of celecoxib for 4 days and cell viability was assessed using a Sulforhodamine B (SRB) assay. As shown in Fig. 6a, b, gene deletion of *LAMC2*, *MFGE8*, *KLK5*, *KLK7*, or *SLC2A1* all significantly increased sensitivity to celecoxib, resulting in a lower IC50 value as compared to scrambled cells. Loss-of-function mutations in *TPM4*, *RGS2*, *SERPINB5*, *ID4*, or *RBP1* did not affect sensitivity to celecoxib in MDA-MB-231 cells. These results were further confirmed using a Prestoblue cell viability assay in *LAMC2*, *MFGE8*, *KLK5*, and *SLC2A1* KO cells, treated or not with 50 μM celecoxib for 4 days. As shown in Fig. 6c, while celecoxib led to a 27.3% reduction in cell viability in control cells, this effect was strongly enhanced in *LAMC2*, *MFGE8*, *KLK5*, and *SLC2A1* KO cells (51.5%, 49.3%, 47.9%, and 51.3% reduction in cell viability, respectively), indicating that individual deletion of these genes could sensitize TNBC cells to celecoxib treatment. In order to confirm these findings in a different genetic background and to avoid the limitation of using a single cell line, we knocked out *LAMC2*, *MFGE8*, *KLK5*, *KLK7*, and *SLC2A1* genes in another TNBC cell line (SUM159PT, hereafter referred to as SUM159, derived from a patient with anaplastic breast carcinoma). Similar to what was observed in MDA-MB-231 cells, all KOs, with the exception of *LAMC2*, were able to decrease celecoxib IC50 values (Fig.S6A and B). Moreover, as shown in Fig.S6C, when assessed in the Prestoblue assay, we were able to restore sensitivity to celecoxib in four KOs (*LAMC2*, *MFGE8*, *KLK5*, and *SLC2A1).*

Taken together, these data indicate that silencing the expression of *LAMC2, MFGE8*, *KLK5*, *KLK7*, and *SLC2A1* can increase celecoxib sensitivity in TNBC cells in vitro, yet the underlying mechanisms need to be further investigated.

### Role of COX-2-associated candidate genes MFGE8, KLK5, and KLK7 in regulating celecoxib resistance in vivo

**Fig. 7:**
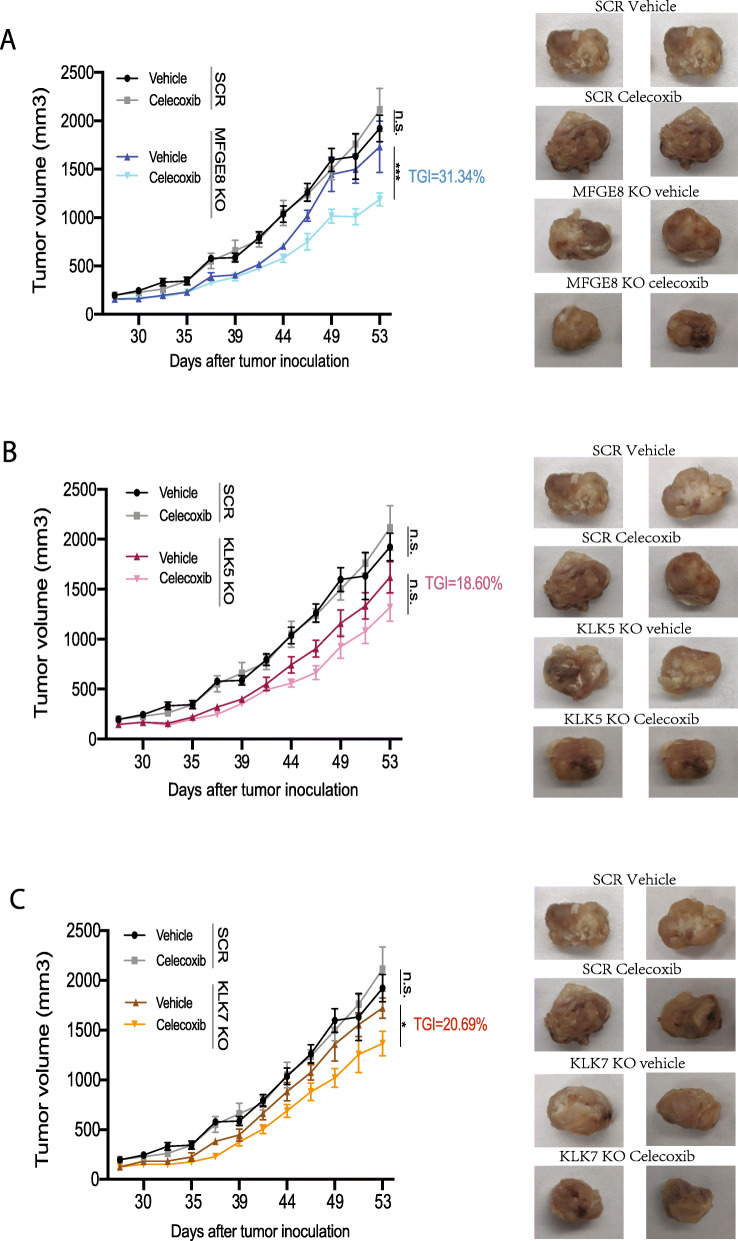
Role of COX-2-associated genes in regulating celecoxib resistance in vivo. Primary tumor growth curves and tumor images from mice inoculated with MDA-MB-231 control, MFGE8 (**a**), KLK5 (**b**), and KLK7 (**c**) KO cells and randomly grouped (*n* = 8/group). Celecoxib treatment was performed as described in the “Methods” section

Having shown that *MFGE8*, *KLK5*, and *KLK7* KO not only substantially suppressed tumor metastasis but also restored celecoxib sensitivity to a greater degree compared to other candidate genes, we next focused on these three genes and tested the efficacy of *MFGE8*, *KLK5*, and *KLK7* individual KO combined with COX-2 inhibition in tumor suppression using an orthotopic xenograft mouse model. Briefly, scrambled, *MFGE8*, *KLK5*, and *KLK7* KO cells generated in the MDA-MB-231 background were transplanted into the mammary fat pad of NSG mice (1 × 10^6^ cells per mouse) and allowed for orthotopic tumor growth. Mice injected with scrambled and gene KO cells were randomly divided into two groups and treated with vehicle or celecoxib (7.5 mg/kg/day) through IP injection for up to 4 weeks with weight and tumor volume assessed three times weekly. As shown in Fig. 7a–c, when using a suboptimal dosage of celecoxib that did not lead to tumor size reduction in control mice (vehicle vs celecoxib in scrambled KOs, black and gray lines, respectively), we found that deletion of *MFGE8*, *KLK5*, and *KLK7* all resulted in a significant restoration of the celecoxib effect and markedly decreased tumor size by 31.3%, 18.6%, and 20.7%, respectively, following celecoxib treatment compared to the vehicle-treated mice (Fig. 7a–c, colored lanes).

To investigate whether the expression levels of *MFGE8*, *KLK5*, and *KLK7* are complementarily upregulated when COX-2 pathway is inhibited, we generated two celecoxib-resistant MDA-MB-231 cell line by treating the parental cells with 40 and 80 μM celecoxib for 3 weeks.

As shown in Fig.S7A, we found COX-2 to be over-expressed in celecoxib-resistant cell line compared with their parental counterparts, which is consistent with a previous study where COX-2 was overexpressed during the selection of celecoxib-resistant clones in aggressive breast cancer cell lines [28]. This was further verified at the protein levels using immunoblot analysis (Fig. S7B). This also suggests that COX-2 might serve as a contributor to celecoxib resistance in certain contexts. However, when assessing expression of our 10 shortlisted genes, only LAMC2, besides COX-2, was found to be significantly upregulated in the celecoxib-resistant cells (Fig.S7A). None of the other genes were significantly altered in the resistant cell line (data not shown).

The differences between our results and the analysis from CTRP data might be due to various mechanisms of action of different COX-2 inhibitors. Based on these data, our interpretation is that the other 9 genes (*TPM4*, *RGS2*, *LAMC2*, *SERPINB5*, *KLK7*, *MFGE8*, *KLK5*, *ID4*, *RBP1*, *SLC2A1*) are neither downstream targets of COX-2 signaling pathway nor complementary pathways upregulated in COX-2 inhibitor-resistant cells. They are correlated with COX-2 expression in TNBC patients given our selection method of these candidate genes and the data shown in Fig. S5. In addition, we hypothesize that the potential synergistic effects of COX-2 inhibition and some genes KO (KLK5/7, MFGE8) on suppressing TNBC primary tumor growth might be due to convergent inhibition of different aspects of tumorigenesis.

Although more underlying mechanisms need to be further illustrated, these data suggest that gene deletion of *MFGE8*, *KLK5*, or *KLK7* can sensitize TNBC to celecoxib in preclinical model, providing a rationale for targeting these genes in combination with COX-2 inhibitors for celecoxib-resistant TNBC. Although we cannot predict whether these will be sufficient to produce a change in a clinical setting, these encouraging results suggest that using clinical scenarios targeting all (or some of) the identified genes simultaneously have the potential to further increase any associated clinical benefits for TNBC treatment.

Collectively, through in vitro and in vivo function validation experiments, we found that deletion of three genes (*MFGE8*, *KLK5/7*) can reduce TNBC lung metastasis and simultaneously increase sensitivity to COX-2 inhibitors in vivo. The observed cooperative effects of blocking *MFGE8* and *KLK5/7* with COX-2 inhibition in reducing tumor growth provides an important rationale for developing COX-2 inhibitor-based combination therapies for breast cancer patients.

## Discussion

COX-2 is overexpressed in 40% of cases of invasive breast carcinoma and has been implicated in multiple steps during breast tumor progression, including primary tumor growth, metastasis, angiogenesis, and immune evasion. Even though COX-2 inhibitors have been proven to attenuate breast tumor growth and metastasis in preclinical models, the clinical benefit of COX-2 inhibitors in breast cancer patients remain elusive. Meta-analyses of aspirin use showed a 9–30% reduced risk of breast cancer incidence [34]. Regular use of COX-2 inhibitors was also associated with 60–70% reduced risk of breast cancer for women at familial or genetic risk [35]. Celecoxib (Celebrex) has been approved by FDA to treat arthritis patients, and its potential to treat cancer patients such as breast cancer is still under investigation. A clinical study in breast cancer showed that pre-operative celecoxib treatment sets up transcriptional programs supporting anti-tumor activity [36]. Other trials demonstrated that combination of celecoxib with aromatase inhibitors in the neoadjuvant treatment is effective in reducing breast tumor size and area [23, 24]. Results from a randomized phase II trial of celecoxib plus exemestane compared with exemestane alone in patients with hormone-sensitive breast cancer (*n* = 111) suggested a trend in favor of combination therapy, evidenced by an approximately twofold longer duration of clinical benefit in patients receiving the combination treatment [37]. However, a phase III multicenter double-blind randomized trial of celecoxib versus placebo in primary breast cancer patients showed no benefit of celecoxib in BC patients [25]. Several possibilities might explain the mixed results produced by these studies. First, there were no proper stratification criteria established for breast cancer patients receiving COX-2 inhibitor treatment. Also, little is known regarding mechanisms for underlying COX-2 inhibitor insensitivity/resistance in breast cancer. Thus, the aim of our study is to characterize the role of COX-2 and COX-2-associated genes in regulating breast cancer tumorigenesis as well as to identify COX-2 inhibitor resistance genes. We mainly focused on an extremely aggressive subtype of breast cancer, TNBC in this study, since patients with TNBC exhibit poor prognosis and lack of specific actionable molecular targets. Leveraging existing large breast cancer databases and cohorts with genomic and transcriptomic profiles as well as clinical data, we were able to develop a systematic data mining strategy to identify COX-2-associated genes in TNBC that are correlated with its aggressive features and breast cancer resistance to COX-2 inhibitor. Using CRISPR/Cas9 gene editing tools and preclinical models of breast cancer, we functionally validated the identified genes and addressed their roles and contributions to (1) breast cancer metastasis and (2) breast tumor resistance to COX-2 selective inhibitor, celecoxib. We found 10 genes (*TPM4*, *RGS2*, *LAMC2*, *SERPINB5*, *KLK7*, *MFGE8*, *KLK5*, *ID4*, *RBP1*, *SLC2A1*) that regulate TNBC distant lung metastasis in vivo, among which 6 genes (*TPM4*, *RGS2*, *SERPINB5*, *MFGE8*, *KLK5*, *ID4*) individual KO led to more than 90% reduction of lung metastatic area. We also showed that individual knockouts of *MFGE8*, *KLK5*, and *KLK7* resulted in increased sensitivity to celecoxib in TNBC both in vitro and in vivo. These results demonstrate the robustness and power of our multi-level in silico data analysis strategy combined with in vitro*/*in vivo functional validation. This systematic approach could be applied to other studies with the goal of investigating any gene/pathway-associated gene network and their regulation in any specific step of tumorigenesis as well as mechanisms of acquired drug resistance. We found that *MFGE8* gene KO led to more than 90% reduction of TNBC lung metastatic area in our preclinical model. *MFGE8* gene encodes for the milk fat globule-EGF factor 8, a secreted glycoprotein that mediates adhesion to integrin-expressing cells [38]. *MFGE8* has been shown to regulate tumorigenesis through multiple mechanisms: enhancing phagocytosis of apoptotic cells by endothelial and epithelial cells [39, 40]; inducing tumor mesenchymal phenotype through the activation of Akt [41]; as well as promoting vascular endothelial growth factor (VEGF)-induced angiogenesis by binding to avb3/b5 integrins [42]. Since no changes of cell phenotype and proliferation rate were observed upon *MFGE8* deletion in TNBC cell lines, we hypothesize that impaired *MFGE8*-mediated angiogenesis might be the mechanism underlying reduced lung metastasis in *MFGE8* KO tumors. Interestingly, COX2/PGE2 pathway was found to regulate tumor angiogenesis in a VEGF-independent manner and mediate refractoriness to VEGF/VEGFR2 inhibition [43]. Similar observations were made by another research group, showing that COX-2 inhibition improves the efficacy of antiangiogenic therapy in breast cancer and colorectal cancer preclinical models [44]. Since MFGE8 was not upregulated in celecoxib-resistant TNBC cells, the potential synergistic effects of MFGE8 deletion with COX-2 inhibition on suppressing TNBC primary tumor growth might be due to convergent anti-angiogenesis pathway rather than overcoming celecoxib resistance and should be investigated in future studies. Our results also suggest that combination treatments aiming at disabling both COX-2 and MFGE8 could represent a therapeutic strategy for the treatment of TNBC. Although MFGE8 has been shown to be overexpressed in TNBC compared with non-TNBC patients [45], it is also an essential gene for the breast involution process [46]. Thus, precise examination and attentive care will be required when targeting MFGE8 in clinical settings to avoid any potential side effects related to abnormal mammary gland remodeling.

Kallikrein-related peptidase 5 and 7 (KLK5 and KLK7) are members of a subgroup of 15 homologous secreted serine proteases and are highly expressed in endocrine or hormone-responsive tissues including breast, ovary, and skin [47, 48]. KLK5 has been shown to activate KLK7 in vitro and is considered as the physiological activator of KLK7 [49]. Several studies have shown that KLK5 and KLK7 serve as serological biomarkers and indicators of poor prognosis in breast and ovarian cancer [50–54]. Consistent with these findings, we found high expression of KLK5 and KLK7 to correlate with aggressive pathological features and poor patient outcomes in TNBC. In addition, kallikrein-regulated extracellular proteolysis is implicated in many cancer-related processes, such as tumor cell growth, invasion, metastasis, and angiogenesis [55]. Indeed, KLK5 has recently been shown to cleave ECM (collagens type I, II, III, IV, fibronectin, and laminin) and adhesion molecules (fibrinogen and vitronectin), suggesting a role in tumor invasion and angiogenesis [47]. In our study, both *KLK5* and *KLK7* gene KOs in TNBC cell lines block distant lung metastasis in vivo, demonstrating their pro-tumorigenic function. Moreover, as a COX-2 associated gene in TNBC, we found *KLK5* and *KLK7* gene KOs to restore tumor cell sensitivity to celecoxib both in vitro and in vivo. Although there is no literature showing the direct interactions between KLK and COX-2 signaling pathway, the cooperativity between COX-2 inhibition and KLK KO in reducing tumor growth is worth further investigation. A better understanding of the crosstalk between the COX-2 pathway and KLK pathways will be useful for future design and personalization of novel COX-2 inhibitor-based combination therapies in clinical settings. The potential of KLK5 and KLK7 as therapeutic targets in cancer has led to advances in the development of the first generation of KLK-based inhibitors. As of current, these pharmacological efforts are mainly directed toward the design of small-molecule inhibitors, such as triazole derivatives [56] and other compounds identified in high-throughput screening of large chemical libraries as well as peptide/protein-based inhibitors [57–59]. Thus, it will be interesting to further test the use of the various KLK inhibitors in TNBC, using combi-therapy with the FDA-approved anti-COX-2 drugs.

## Conclusions

Combined in silico data analysis, in vitro and in vivo function validation provided meaningful insights into strategies to restore sensitivity to COX-2 inhibitor. Furthermore, having identified MGFE8 and KLK5/7 as key promoters of breast tumorigenesis, our study supports the establishment of novel COX-2 inhibitor-based combination therapies as a future strategy for TNBC treatment.

## Supplementary Information

**Additional file 1:.** Figure S1. DEGs in COX-2-high patient and COX-2-low patient groups.

**Additional file 2: **Figure S2. a, Percentage of gene copy number amplification (orange) and mRNA upregulation (grey) of each of the 43 DEGs in metastatic breast cancer patient cohorts (*n* = 180). Genes to the left of the dashed lines were considered as highly amplified and expressed. b, mRNA expression levels of final selected 10 candidate genes in TNBC versus non-TNBC patients in a collection of breast cancer patient cohort from the Breast Cancer Gene-Expression Miner v4.0 online platform.

**Additional file 3: **Figure S3. Kaplan-Meier survival analysis showing the relationship between each of the candidate gene expression and overall survival as well as distant metastasis free survival rates in a collection of basal breast cancer patient cohorts (*n* = 241) from Kaplan-Meier plotter online platform.

**Additional file 4:.** Figure S4. mRNA expression levels of 10 candidate genes across various sample types (primary tumor, metastatic tumor, solid normal tissue) in TCGA dataset.

**Additional file 5:.** Figure S5. Pearson's correlation analysis of 10 candidate genes expression with COX-2 expression in TNBC patients from TCGA dataset.

**Additional file 6:.** Figure S6. a, SUM159 control and KO cell lines were treated with a dose rage of celecoxib for 4 days and subjected to cell viability test using SRB assay. IC50 values of control and each KO cells were indicated. b, Celecoxib dose response curve in SUM159 control, LAMC2, KLK7, MFGE8, KLK5, and SLC2A1 KO cells. c, SUM159 control, LAMC2, MFGE8, KLK5, and SLC2A1 KO cells were treated with 50 μM celecoxib for 4 days and then subjected to Prestoblue cell viability assay. Percentage of living cells after celecoxib treatment was calculated.

**Additional file 7:.** Figure S7. a, mRNA expression levels of COX-2 and LAMC2 were assessed in MDA-MB-231 parental cells and two celecoxib-resistant variant cell lines by qPCR. b, COX-2 protein levels were measured in MDA-MB-231 parental cells and celecoxib-resistant cells by western blot.

**Additional file 8.** CRISPR/Cas9 sgRNA sequences targeting 10 COX-2 associated genes.

**Additional file 9.** qPCR Primers for 10 COX-2 associated genes.

## Data Availability

All data generated or analyzed during this study are included in this published article and its supplementary information files.
