# Supplementary material for: Identification of MFGE8 and KLK5/7 as mediators of breast tumorigenesis and resistance to COX-2 inhibition
Source: Breast Cancer Res. 2021 Feb 15;23:23. doi: 10.1186/s13058-021-01401-2 (PMC7885389; doi:10.1186/s13058-021-01401-2)
Supplement: Supplementary file 1 — Additional file 1:. Figure S1. DEGs in COX-2-high patient and COX-2-low patient groups. [file 13058_2021_1401_MOESM1_ESM.pdf]

| *Patient group* | *Gene symbol* |
|---|---|
| High expression in COX-2-high TNBC patients | TPM4, PTGS2, TNFRSF21, RGS2, TFAP2C, ITGA6, DEGS1, ATP1B1, COL9A2, SFRP1, FURIN, LAMC2, THBS1, CD55, TACSTD2, MSN, S100B, CTNNB1, FBXO32, STAC2, DSP, KRT6B, FBLN2, SERPINB5, IER3, ITGB4, FOXI1, EPHB3, MDFI, KLK7, OGFRL1, MFGE8, ACTN4, KLK5, SLC44A2, KLK6, ID4, RBP1, PTP4A1, TMX4, SLC2A1, GNAS, PPP1R1B |
| High expression in COX-2-low TNBC patients | MRPL51, MRPS35, CST3, RLP41P1, ALDH3B2, FASN, RARRES3, IDH2, RN7SL2, AC090498.1, UBA52, CENPX, MRPL20, PRDX3, CFD, GGCT, PIP, COX6A1, TSTA3, SLC25A6, FTL, PTMS, NDUFB7, DDX49, DCHS2, AURKAIP1, RPLP0, UCP2, TFAP2B, AC093001.1, AHCY, ISYNA1, SPDEF, APOC1, ECHS1, TEF3, RPS5, PHB2, RPL21, FAU, NOP53, COPE, NHP2, RPS4X, RPL18A, C12orf57, APOE, RPL22, WDR34, RPS3, RACK1, EIF4EPB1, USP5, RPS13, RPL36AL, RPL41, ATP5PF, PRR15L, RPS29, BSPRY |

Figure S1
