# Supplementary figures and images for: Identification of MFGE8 and KLK5/7 as mediators of breast tumorigenesis and resistance to COX-2 inhibition
Source: Breast Cancer Res. 2021 Feb 15;23:23. doi: 10.1186/s13058-021-01401-2 (PMC7885389; doi:10.1186/s13058-021-01401-2)

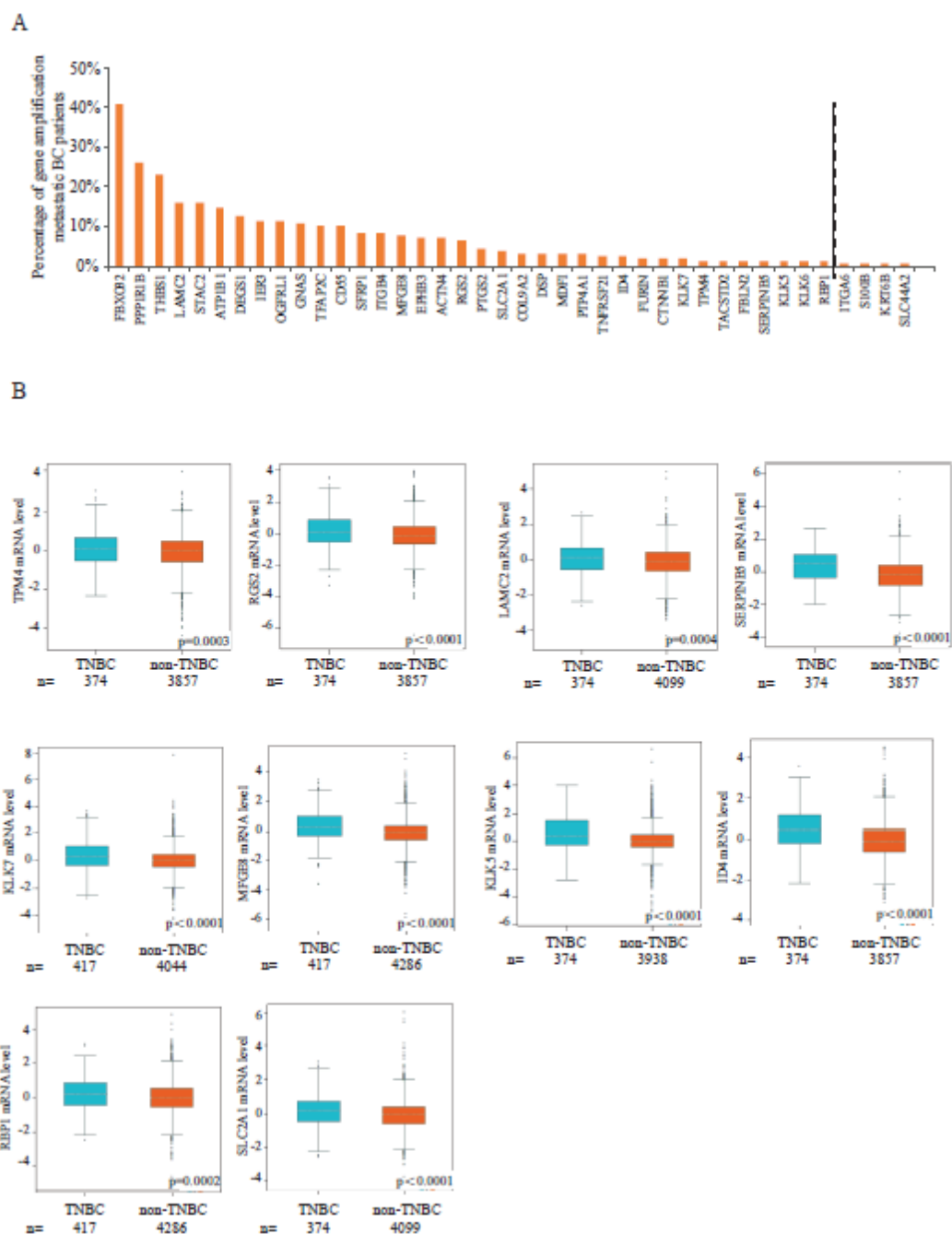

Figure S2

Supplement: Supplementary file 2 — Additional file 2: Figure S2. a, Percentage of gene copy number amplification (orange) and mRNA upregulation (grey) of each of the 43 DEGs in metastatic breast cancer patient cohorts (n = 180). Genes to the left of the dashed lines were considered as highly amplified and expressed. b, mRNA expression levels of final selected 10 candidate genes in TNBC versus non-TNBC patients in a collection of breast cancer patient cohort from the Breast Cancer Gene-Expression Miner v4.0 online platform. [file 13058_2021_1401_MOESM2_ESM.pdf]

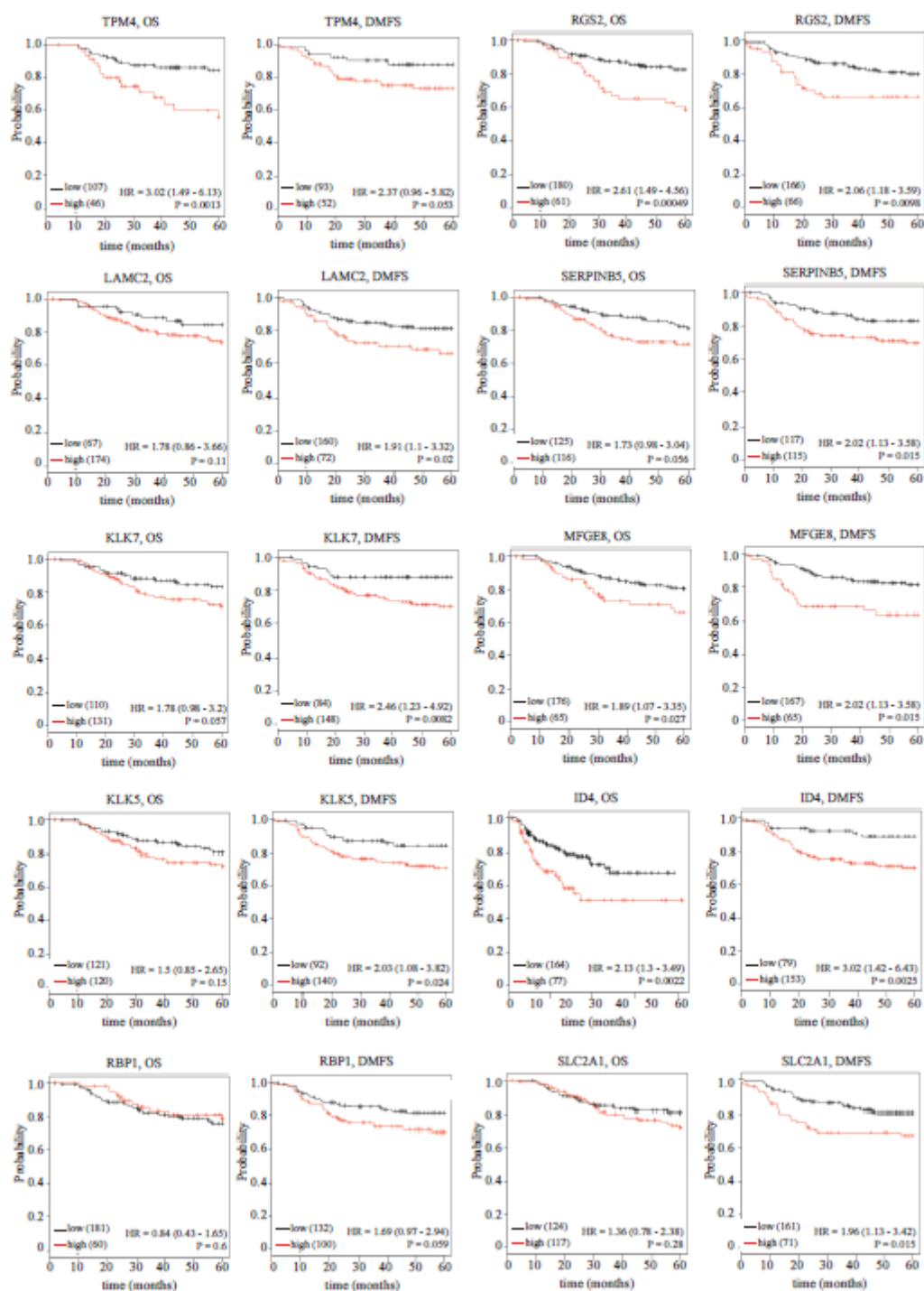

Figure S3

Supplement: Supplementary file 3 — Additional file 3: Figure S3. Kaplan-Meier survival analysis showing the relationship between each of the candidate gene expression and overall survival as well as distant metastasis free survival rates in a collection of basal breast cancer patient cohorts (n = 241) from Kaplan-Meier plotter online platform. [file 13058_2021_1401_MOESM3_ESM.pdf]

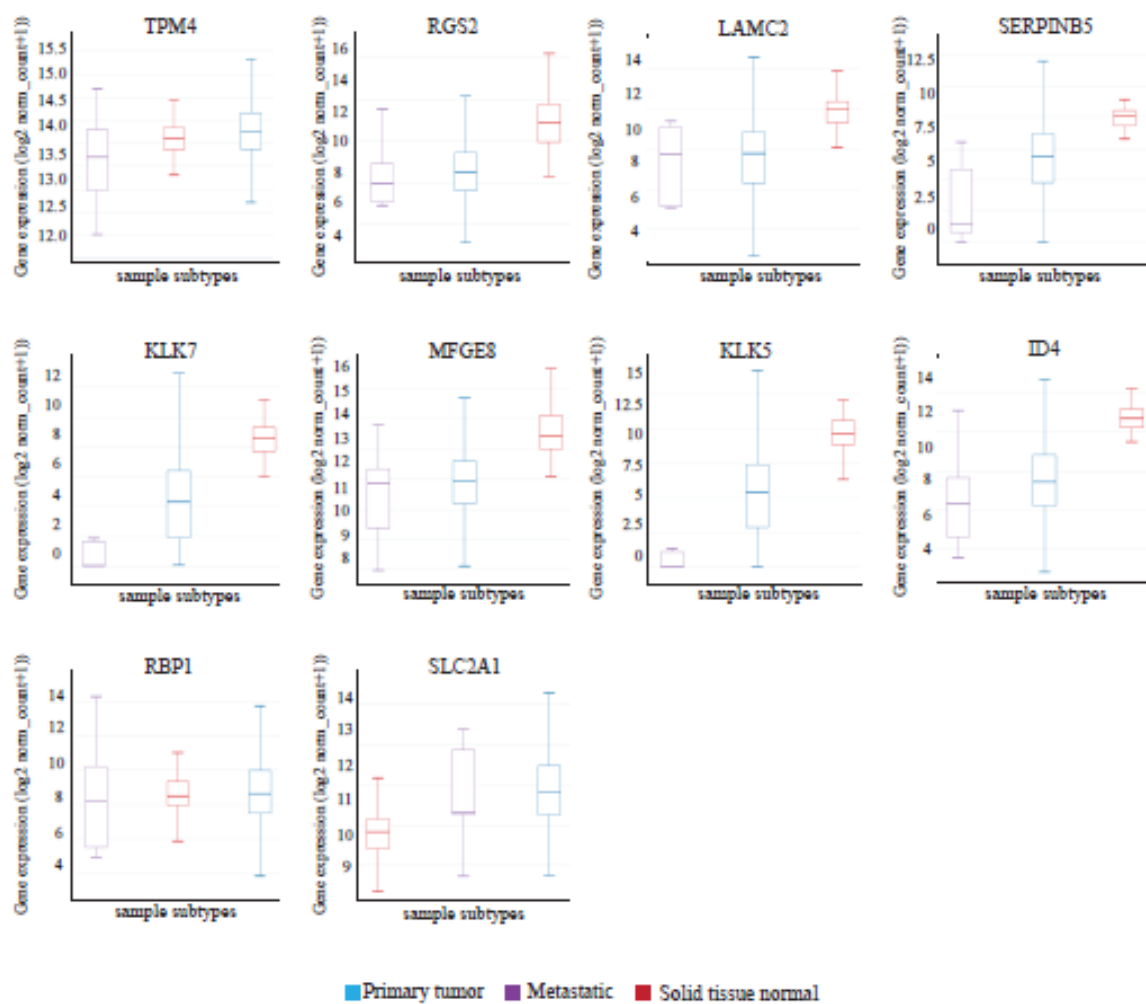

Figure S4

Supplement: Supplementary file 4 — Additional file 4:. Figure S4. mRNA expression levels of 10 candidate genes across various sample types (primary tumor, metastatic tumor, solid normal tissue) in TCGA dataset. [file 13058_2021_1401_MOESM4_ESM.pdf]

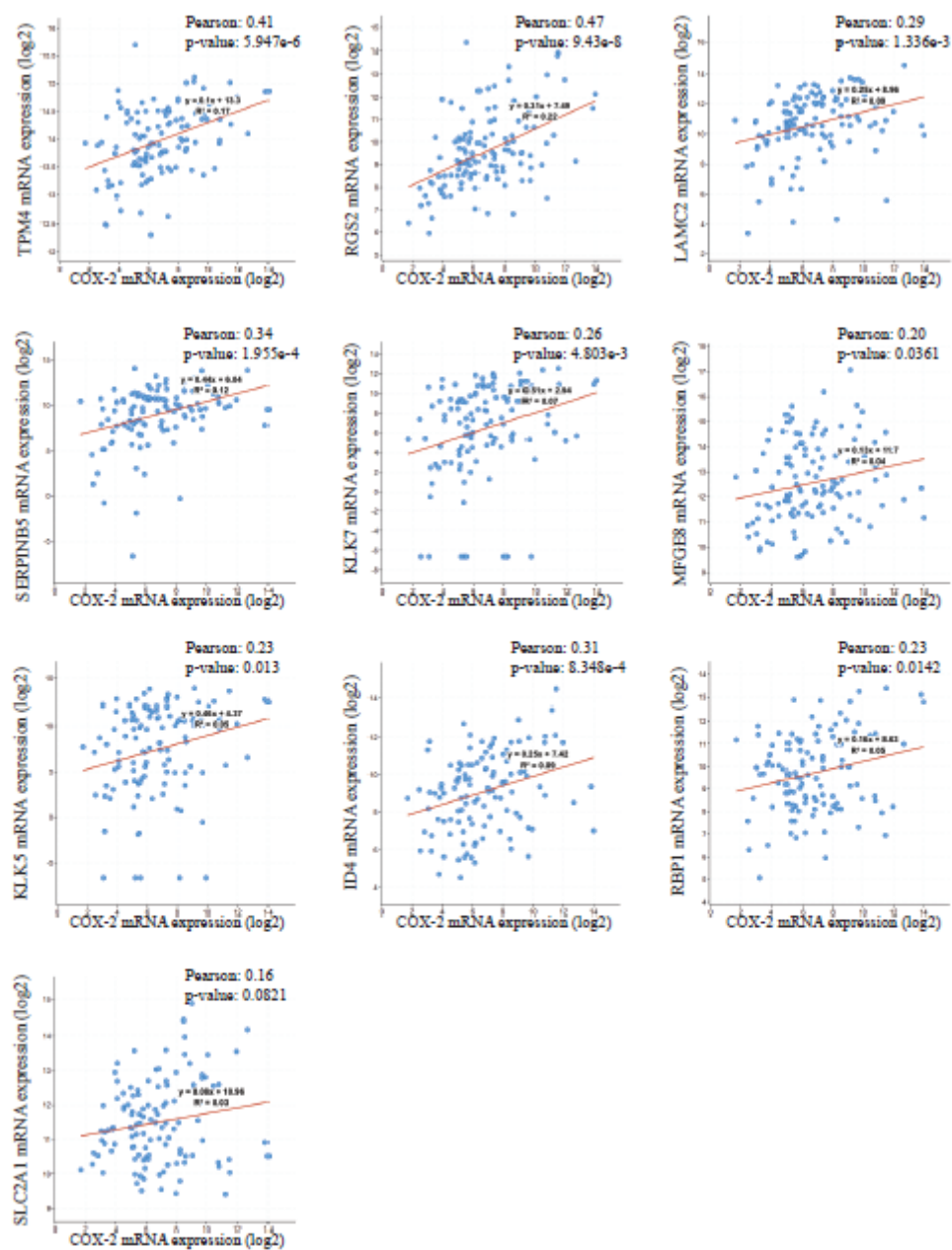

Figure S5

Supplement: Supplementary file 5 — Additional file 5:. Figure S5. Pearson's correlation analysis of 10 candidate genes expression with COX-2 expression in TNBC patients from TCGA dataset. [file 13058_2021_1401_MOESM5_ESM.pdf]

A

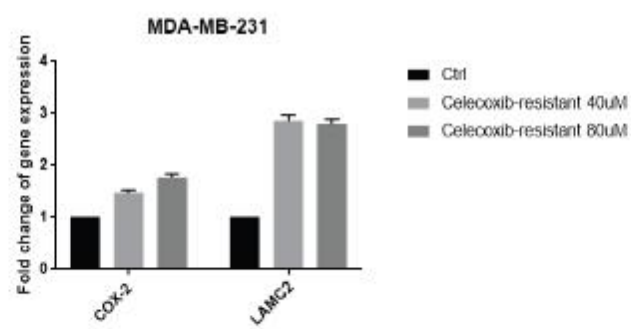

B

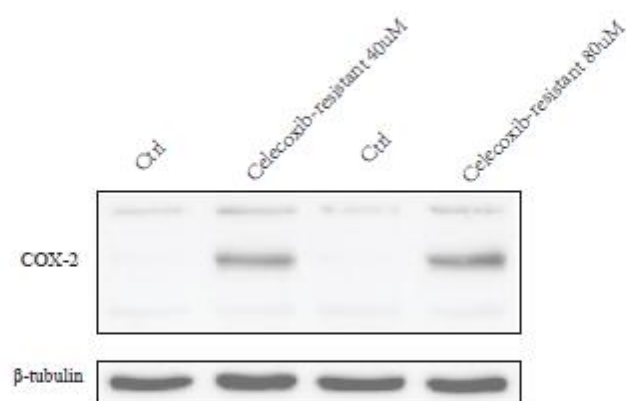

Figure S7

Supplement: Supplementary file 7 — Additional file 7:. Figure S7. a, mRNA expression levels of COX-2 and LAMC2 were assessed in MDA-MB-231 parental cells and two celecoxib-resistant variant cell lines by qPCR. b, COX-2 protein levels were measured in MDA-MB-231 parental cells and celecoxib-resistant cells by western blot. [file 13058_2021_1401_MOESM7_ESM.pdf]
