# Supplementary material for: Identification of MFGE8 and KLK5/7 as mediators of breast tumorigenesis and resistance to COX-2 inhibition
Source: Breast Cancer Res. 2021 Feb 15;23:23. doi: 10.1186/s13058-021-01401-2 (PMC7885389; doi:10.1186/s13058-021-01401-2)
Supplement: Supplementary file 6 — Additional file 6:. Figure S6. a, SUM159 control and KO cell lines were treated with a dose rage of celecoxib for 4 days and subjected to cell viability test using SRB assay. IC50 values of control and each KO cells were indicated. b, Celecoxib dose response curve in SUM159 control, LAMC2, KLK7, MFGE8, KLK5, and SLC2A1 KO cells. c, SUM159 control, LAMC2, MFGE8, KLK5, and SLC2A1 KO cells were treated with 50 μM celecoxib for 4 days and then subjected to Prestoblue cell viability assay. Percentage of living cells after celecoxib treatment was calculated. [file 13058_2021_1401_MOESM6_ESM.pdf]

A

CRISPR knock out in SUM159 cells	*IC50 +/- SEM (uM)*	*P value*
**Scramble**	44.19 +/- 2.05	
**LAMC2**	43.57 +/- 4.29	0.924
**KLK7**	32.5 +/- 0.44	0.026
**MFGE8**	33.01 +/- 1.73	0.017
**KLK5**	35.62 +/- 3.53	0.119
**SLC2A1**	36.68 +/- 0.51	0.068

B

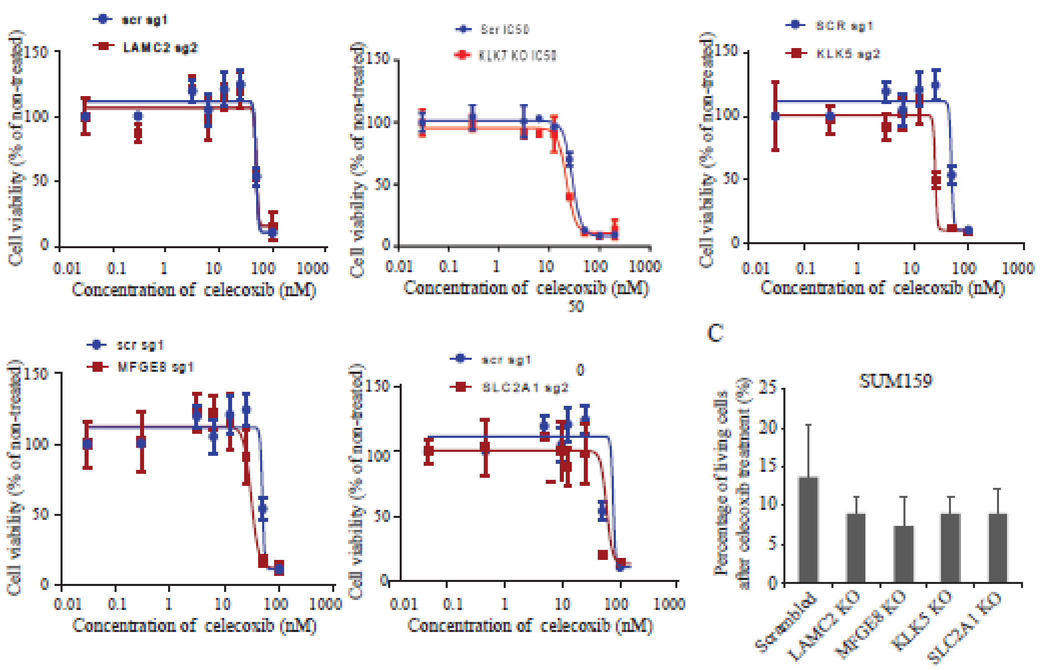

Figure S6
