# Supplementary material for: Identification of MFGE8 and KLK5/7 as mediators of breast tumorigenesis and resistance to COX-2 inhibition
Source: Breast Cancer Res. 2021 Feb 15;23:23. doi: 10.1186/s13058-021-01401-2 (PMC7885389; doi:10.1186/s13058-021-01401-2)
Supplement: Supplementary file 8 — Additional file 8. CRISPR/Cas9 sgRNA sequences targeting 10 COX-2 associated genes. [file 13058_2021_1401_MOESM8_ESM.pdf]

		gRNA sequences
TPM4	sg1	CTCACCAGAGAAACTTGCCC
	sg2	GCTTCTCCTGCGCGTCCTTC
RGS2	sg1	AATTCTGACTCCAAGAAACG
	sg2	CAGATGGTCTTGCTGCATTC
LAMC2	sg1	TGGCAGACATCGGTCGCATC
	sg2	AGTGCTCGATGTGACAACTC
SERPINB5	sg1	CGACCAGACCAAAATCCTTG
	sg2	ATTTGATAGGGCCACTCCCT
KLK7	sg1	GCCGCAAGGGAAAGTTCCCC
	sg2	AGGGTACCTCTGCACACCAA
MFGE8	sg1	TGACCATGCCTGCGCGGTTC
	sg2	GTAGCCCTTAAGGCACGTGC
KLK5	sg1	GGCCGGGGAAGACGCCCGGT
	sg2	ACAGGGAGTAGTGGCCGAGA
ID4	sg1	GGCCGGCGCGGTGAACAAGC
	sg2	CACTGCGCTCAACACCGACC
RBP1	sg1	GCTCATCACCCTCGATCCAC
	sg2	CCCCACCGCAGACGTCAATG
SLC2A1	sg1	CCTCGTTGCGGTTGATGAGC
	sg2	CTTCGTGTCCGCCGTGCTCA

Supplementary Table 1
