# Supplementary material for: Identification of MFGE8 and KLK5/7 as mediators of breast tumorigenesis and resistance to COX-2 inhibition
Source: Breast Cancer Res. 2021 Feb 15;23:23. doi: 10.1186/s13058-021-01401-2 (PMC7885389; doi:10.1186/s13058-021-01401-2)
Supplement: Supplementary file 9 — Additional file 9. qPCR Primers for 10 COX-2 associated genes. [file 13058_2021_1401_MOESM9_ESM.pdf]

| Gene name | | Forward primer | Reverse primer |
|---|---|---|---|
| TPM4 | sg1 | AAGATCGCGCCACTGCACTC | GAGAGCTGAAAAAGCTGGTGCC |
| | sg2 | CGCCTTCTCCTCCTCCTCTT | TGGTCCTCAGGACGAGGGAA |
| RGS2 | sg1 | CAAGGTCAGTCTTTATGGCAGGTC | AGGAGGGGTAAAAAGTCCCTCCA |
| | sg2 | CTAGCCCGCTTTGTCCTTGATTAC | CAGGTGGGGAAGAAAATCAGCC |
| LAMC2 | sg1 | GGTGATAGTTGCTTCCAATGCCG | CTCACCGGTAACATCAGGCAAC |
| | sg2 | GCAGCAGATGGTGCTTCTTACTTC | TCCCAACCTCTTAGGGGTATTGTG |
| SERPINB5 | sg1 | ATGCCCCACTCTGTCCCTATC | CTTCCTTTCTTCCCTGCTCCTTC |
| | sg2 | ACCATGGCCAATGCCAAGGTC | GGCTTCCTGATCCAGCAACATTAG |
| KLK7 | sg1 | GCGTCCTCACTCCTGTGCAT | CCCCTTCTCTGCAATTGGTCTC |
| | sg2 | AACTCAGTGTGGCGTTAGCGATG | GGAGCAGGGTCCTTAACATTGG |
| MFGE8 | sg1 | AACCCCAGTGATGAACCCTCC | TGCCTTCATTGTCCCTTTGTGGTC |
| | sg2 | TCTCACCTGTGTCCCCAGCT | TGCATCAGCATCAGGCCTGG |
| KLK5 | sg1 | ACTTCTTCCTGCAGTGGGCG | TACCTGAGCCTGGGCTCTGT |
| | sg2 | GGGTCTGACATCTTTAGTGGGACG | GGTGGGGTTGGAGATGGTTG |
| ID4 | sg1 | TGCTCTCAGAAACGCTGGGG | TCAACACCGACCCGGTGAGA |
| | sg2 | CGTTATCGACTACATCCTGGACC | TCCTCGGAAATCAGGCTGGC |
| RBP1 | sg1 | TATCAAGTTTGGGAGCTGCCCCCT | GCAAAAGGGCTTAGCTCATTGCTG |
| | sg2 | TCTATGCCTGTCAGATCCTCCTC | ATGTCTGCTCGTTGGCCCTG |
| SLC2A1 | sg1 | TGTCCCGCGCAGCTTCTTTAG | CATGTGACCGATGAGGAAACTGAG |
| | sg2 | GTGGGAGGTAGGGGAGACTT | TGGGCGGAAGAGAAACTCTGC |

Supplementary Table 2
